# Immunomodulatory Effect of *Flammulina rossica* Fermentation Extract on Healthy and Immunosuppressed Mice

**DOI:** 10.3390/molecules28155825

**Published:** 2023-08-02

**Authors:** Yingdi Dai, Sijia Ma, Yanyan Zhu, Andrey A. Gontcharov, Yang Liu, Qi Wang

**Affiliations:** 1 Engineering Research Center of Chinese Ministry of Education for Edible and Medicinal Fungi, Jilin Agricultural University, Changchun 130118, China; daiyingdi@mails.jlau.edu.cn (Y.D.); m13633954863@163.com (S.M.); 13610712780@163.com (Y.Z.); 2 College of Plant Protection, Jilin Agricultural University, Changchun 130012, China; 3 Institute of Biology and Soil Science, FEB RAS, 100-Letia Vladivostoka Prospect, 159, Vladivostok 690022, Russia; gontcharov@biosoil.ru

**Keywords:** fermented extract, *Flammulina rossica*, liquid fermentation, immunomodulatory, T lymphocytes

## Abstract

*Flammulina rossica* fermentation extract (FREP) was obtained by ethanol precipitation of the fermentation broth. The molecular weight of FREP is 28.52 kDa, and it mainly contains active ingredients such as polysaccharides, proteins, reducing sugars, and 16 amino acids. Among them, the polysaccharides were mannose, glucose, galactose, arabinose, and fucose and possessed β-glycosidic bonds. Furthermore, the immunoregulatory activities of FREP were investigated in vivo. The results demonstrated that FREP could increase the counts of CD^4+^ T lymphocytes and the ratio of CD^4+^/CD^8+^ in a dose-dependent manner in healthy mice. In addition, FREP significantly increased serum cytokines, including IL-2, IL-8, IL-10, IL-12, IL-6, IL-1β, INF-γ, C-rection protein, and TNF-α, and promoted splenocyte proliferation in healthy mice. Finally, FREP could restore the counts of white blood cells, red blood cells, secretory immunoglobulin A, and antibody-forming cells and significantly promote the serum haemolysin level in mice treated with cyclophosphamide. The findings indicated that FREP possessed immunoregulatory activity in healthy mice and could improve the immune functions in immunosuppressive mice. Therefore, FREP could be exploited as an immunomodulatory agent and potential immunotherapeutic medicine for patients with inadequate immune function.

## 1. Introduction

Medicinal or edible mushrooms have attracted increasing scientific attention due to their advantages of improving human health and preventing diseases [1,2]. Many mushrooms have been used as therapeutic agents in various countries [3]. Many bioactive compounds have been found in edible and medicinal mushrooms, including polysaccharides, proteins, peptides, lipids, and sterols [4]. Among the bioactive ingredients, polysaccharides have few side effects and low toxicity. Many polysaccharides derived from mushrooms, including *Dictyophra indusiate*, *Ganoderma lucidum*, *Lentinus edodes*, and *Agaricus brasiliensis*, have multiple pharmacological actions, such as antitumor, immunomodulatory, and antioxidant activities [5,6,7]. Among these activities, immunomodulatory activity has been widely studied. Many studies have indicated that fungal polysaccharides exhibit immunomodulatory effects and regulate the function of a variety of immune cells, such as macrophages, T cells, B cells, dendritic cells, and natural killer (NK) cells [8,9].

Although many edible mushroom polysaccharides, including those from *Flammulina velutipes*, *Cordyceps militaris,* and *Agaricus bisporus*, have been reported to have immunomodulatory activity [10,11,12], many mushroom resources have not yet been developed and utilized. *Flammulina rossica*, an edible basidiomycetous fungus, was discovered in the eastern part of Russia; it belongs to Basidiomycotina, Agaricomycetes, Agaricales, and *Flammulina* and is a newly recorded species in China [13]. To our knowledge, there are few reports on the biological activity of *Flammulina rossica*, and its active components have not been fully studied. Therefore, it is of great significance to develop the pharmacologically active components of *Flammulina rossica*. In our previous study, the fungus *Flammulina rossica* was collected from Shanri-La County in Yunnan, where the altitude was approximately 3000 m, and identified as *Flammulina rossica* by molecular identification (ITS sequence alignment results are provided in the Supplementary Data Figure S1) [14].

The aim of the present research is to investigate the immunomodulating activity of a fermentation extract from *Flammulina rossica* in mice. The fermentation extract was obtained from the fermentation broth of *Flammulina rossica*, and its immunomodulating activities were systematically studied in healthy and immunosuppressed mice.

## 2. Results and Discussion

### 2.1. Physicochemical Properties of the Fermentation Extract FREP

**Figure 1:**
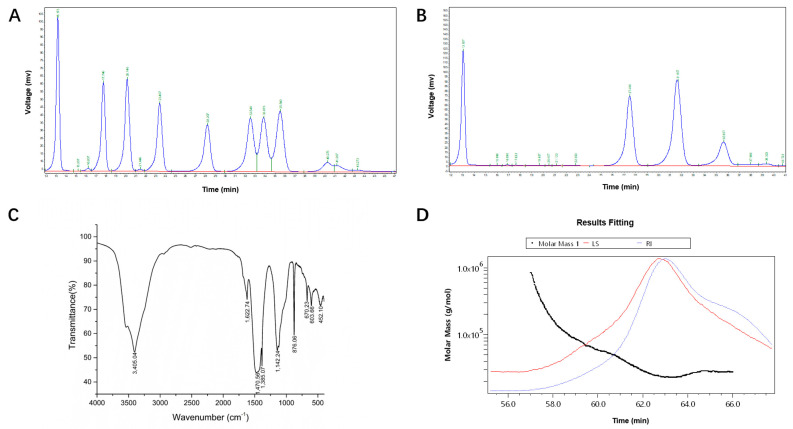
Structural characterization of fermentation extract FREP. (**A**) Ion chromatogram of the standard; (**B**) FREP polysaccharides ion chromatogram; (**C**) FTIR spectra of FREP polysaccharides; and (**D**) molecular weight of FREP polysaccharides.

The main nutritional components of the FREP powders are shown in Table 1. The yield of the FREP was 20.89 g/L. The contents of total carbohydrates, total protein, reducing sugar, and crude fat were 40.5%, 10.40%, 16.67%, and 0.48%, respectively. In addition, 16 types of amino acids were found, and glutamic acid was the most common (Table 2). The FREP consisted of mannose, glucose, galactose, arabinose, and fucose with mole percentages of 22.40%, 26.50%, 37.70%, 12.30%, and 1.10%, respectively (Figure 1B, Table 1). The fourier transform infrared spectroscopy (FTIR) spectrum of FREP was determined, as shown in Figure 1C, and the broad peak at 3405.04 cm^−1^ was assigned to O-H stretching vibrations [15]. The absorption peak at 1622.74 cm^−1^ is the asymmetric stretching vibration absorption peak of C=O [16]. The band at 1470.56 cm^−1^ was due to the deformation vibration absorption peak of C-H. The peak at 1385.07 cm^−1^ was attributed to the C-H deformation vibration. The peaks at 800–1200 cm^−1^ represented the area of carbohydrates [17]. The single peaks at 1142.24 cm^−1^ were due to the stretching vibration of C-O-C linkages and the pyranoid ring [18]. The absorption peak near 900 cm^−1^ was the characteristic peak of the β-glycosidic bond, and the peak near 770 cm^−1^ was the symmetrical ring vibration absorption peak of pyranose. In addition, the molecular weight of FREP was determined by gel permeation chromatography-refractive index-multi angle laser light scattering (GPC-RI-MALS). Figure 1D and Table 1 show that the molecular weight of FREP was 28.52 kDa.

### 2.2. Effect of FREP on the Immune Organ Index in Mice

**Figure 2:**
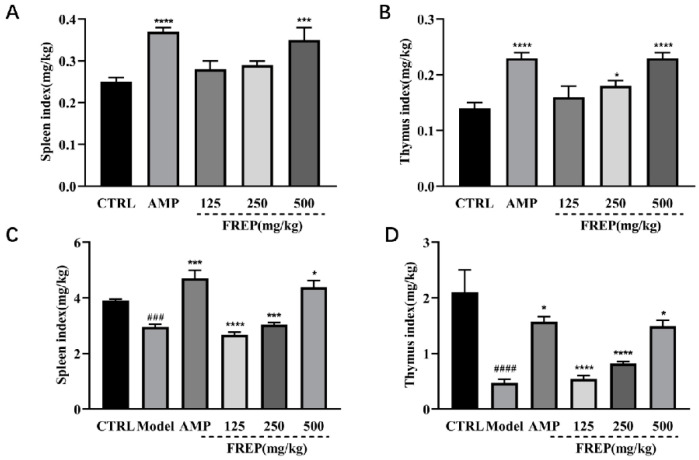
Effects of FREP on immune organ index in mice. (**A**) Spleen index in healthy mice; (**B**) thymus index in healthy mice; (**C**) spleen index in immunosuppressive mice; and (**D**) thymus index in immunosuppressive mice. The data were analyzed using a one-way ANOVA and they are expressed as means ± SEMs. ### *p* < 0.001 and #### *p* < 0.0001 in comparison with the control group (**C**,**D**); * *p* < 0.05, *** *p* < 0.001, and **** *p* < 0.0001 as compared with the control group (**A**,**B**). * *p* < 0.05, *** *p* < 0.001, and **** *p* < 0.0001 as compared with the model group (**C**,**D**). CTRL: normal control; Model: model group; AMP: *Astragalus membranaceus* polysaccharides; and FREP: *Flammulina rossica* fermentation extract.

The effect of FREP on the immune organ index in healthy mice is shown in Figure 2A,B. Compared with the normal group, the positive group had significantly increased thymus and spleen indices (*p* < 0.0001), the medium-dose FREP groups had increased thymus indices (*p* < 0.01), and the high-dose FREP groups had increased thymus and spleen indices (*p* < 0.0001, *p* < 0.001). Compared with the normal group, the spleen index of mice was significantly increased by FREP (500 mg/kg) (*p* < 0.001). The effect of FREP on the immune organ index in immunosuppressed mice is shown in Figure 2C,D. Compared with the model group, the difference in the thymus index of mice in different FREP-dose groups was significant, and the difference was significant between the low- and medium-FREP-dose groups (*p* < 0.0001). Compared with the model group, the spleen index of the low-dose and medium-dose FREP groups was significantly different (*p* < 0.0001, *p* < 0.001).

### 2.3. Effect of FREP on Spleen Lymphocyte Proliferation in Healthy Mice

**Figure 3:**
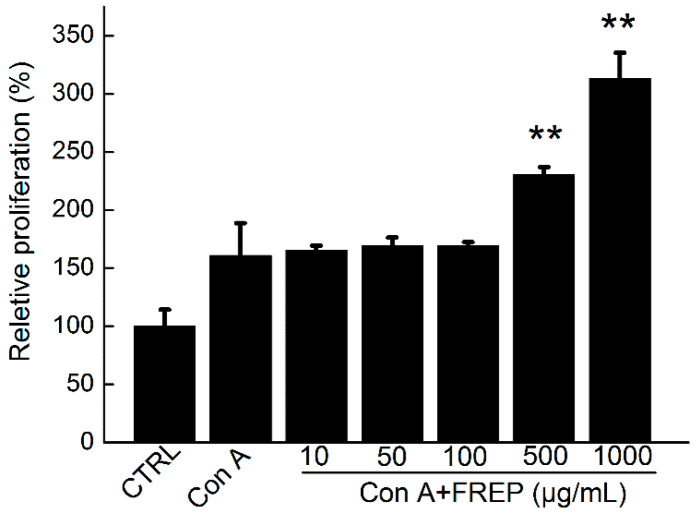
Effect of FREP on splenocyte proliferation of healthy mice in vitro. The data were analyzed using a one-way ANOVA and they are expressed as means ± SEMs (*n* = 8). ** *p* < 0.01 as compared with the normal control group. CTRL: normal control; Con A: concanavalin A; FREP: *Flammulina rossica* fermentation extract.

The effect of FREP on splenocyte proliferation is shown in Figure 3. After stimulation with Con A, the splenocytes of the FREP-treated group possessed stronger proliferation activity than those of the normal group. The high-dose groups (500 and 1000 μg/mL) increased to 130% and 213% of the normal group (*p* < 0.01), respectively. Splenocyte proliferation is an important event related to the immunity improvement of T lymphocytes and B lymphocytes [19,20]. After stimulation, lymphocytes can proliferate and differentiate, leading to a specific cellular immune response [21,22]. The above results suggested that FREP could significantly promote the activation of T and B cells in healthy mice.

### 2.4. Effects of FREP on CD^4+^ and CD^8+^ T Lymphocytes in Healthy Mice

To further investigate the effect of FREP on cellular immunity, the counts of CD^4+^ and CD^8+^ T lymphocytes were measured by flow cytometry. As shown in Table 3, the percentage of CD^4+^ T lymphocytes increased in the high- and low-FREP-dose (500 and 125 mg/kg/high) group (*p* < 0.05) and significantly increased in the medium-FREP-dose (250 mg/kg/high) group compared with the normal control group (*p* < 0.01). The percentage of CD^8+^ T lymphocytes significantly decreased in the high-FREP-dose (500 mg/kg/high) group compared with the normal control group (*p* < 0.01). Moreover, the percentages of CD^4+^/CD^8+^ cells in both the medium- and high-dose groups were higher than those in the normal control group (*p* < 0.01). It is acknowledged that CD^4+^ and CD^8+^ are T helper (Th) and T cytotoxic (Tc) lymphocytes, respectively, which are very important for adaptive immunity [23,24]. Many studies have reported that Th and Tc cells are responsible for releasing proinflammatory cytokines that recruit different effector cells, including macrophages, neutrophils, and eosinophils. In this study, the higher rate of CD^4+^/CD^8+^ cells in the drug groups compared to the normal control group confirmed that FREP may activate the immune system of healthy mice.

### 2.5. Effects of FREP on Serum Cytokines

**Figure 4:**
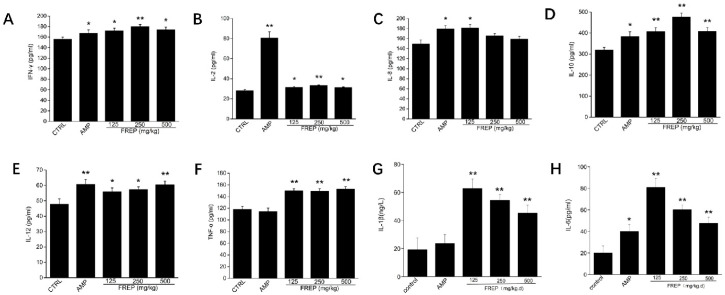
Effects of FREP on serum cytokines. (**A**) IFN-γ levels in the serum of different groups of mice; (**B**) IL-2 levels in the serum of different groups of mice; (**C**) IL-8 levels in the serum of different groups of mice; (**D**) IL-10 levels in the serum of different groups of mice; (**E**) IL-12 levels in the serum of different groups of mice; (**F**) TNF-α levels in the serum of different groups of mice; (**G**) IL-1β levels in the serum of different groups of mice; and (**H**) IL-6 levels in the serum of different groups of mice. The data were analyzed using a one-way ANOVA and they are expressed as means ± SEMs. * *p* < 0.05 and ** *p* < 0.01 as compared with the control group. CTRL: normal control; AMP: *Astragalus membranaceus* polysaccharides; and FREP: *Flammulina rossica* fermentation extract.

The effects of FREP (125, 250, 500 mg/kg/high) on serum cytokines in healthy mice are shown in Figure 4. The results suggested that the production of serum cytokines, including interleukin-2 (IL-2), interleukin-8 (IL-8), interleukin-6 (IL-6), interleukin-1β (IL-1β), interleukin-10 (IL-10), interleukin-12 (IL-12), tumor necrosis factor-α (TNF-α), and interferon-γ (INF-γ), in FREP at various doses was significantly higher than that in the normal control group in healthy mice. It is well-documented that cytokines play vital roles in the immune system and are also potential targets for immunomodulation [25]. Activated Th cells are divided into Th1 and Th2 cells according to their differences and functions. The release of IL-2, IFN-γ, TNF-α, IL-8, IL-1β, IL-6, and IL-12 leads to a Th1 cellular response, whereas Th2 cells secrete IL-10, which is mainly mediated by the humoral immune response. IL-2 is an important cytokine produced by activated T cells and can induce the differentiation and proliferation of T lymphocytes and natural killer cells. TNF-α is mainly secreted by macrophages and induces immune and inflammatory responses [26]. IFN-γ plays a pivotal role in immunoinflammatory reactions and induces an effective immune response against infectious agents and bacteria [27]. Our results showed that FREP was able to significantly increase the levels of serum cytokines in healthy mice, which suggests that FREP possesses the ability to enhance immunity by regulating the secretion of Th1/Th2 cytokines.

### 2.6. Effects of FREP on Haemopoietic Function in Immunosuppressive Mice

**Figure 5:**
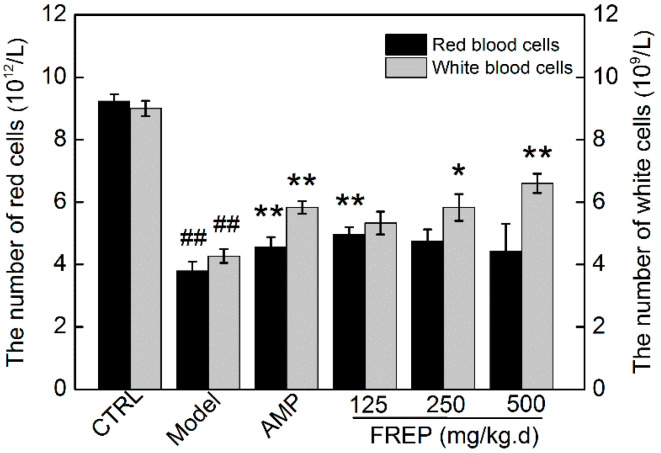
Effect of FREP on the number of red blood cells (RBC, 10^12^/L) and white blood cells (WBC, 10^9^/L) in Cy-treated mice. The data were analyzed using one-way ANOVA and are expressed as means ± SEMs (*n* = 8). ## *p* < 0.01 in comparison with the control group; * *p* < 0.05 and ** *p* < 0.01 as compared with the model group. CTRL: normal control; Model: model group; AMP: *Astragalus membranaceus* polysaccharides; and FREP: *Flammulina rossica* fermentation extract.

To research the protective effect of FREP on the myelosuppression induced by cyclophosphamide, RBCs and WBCs from peripheral blood were determined. As shown in Figure 5, peripheral RBC and WBC counts in Cy-treated mice decreased significantly compared to those in the normal group (*p* < 0.05). However, the counts of RBCs and WBCs were significantly increased by FREP in a dose-dependent manner (*p* < 0.01). The WBC counts in the high-dose group (500 mg/kg/high) were higher than those in the positive group. Previous studies have shown that myelosuppression is an important limiting factor in the outcome and recovery of tumor patients receiving chemotherapy [28]. Our results showed that Cy reduced WBC and RBC counts, and the administration of FREP significantly restored WBC and RBC counts, suggesting that FREP could provide protection against myelosuppression induced by Cy. The results were consistent with previous reports [29]. The number of RBCs was significantly increased in the low-dose FREP group (*p* < 0.01), but not in the medium- and high-dose, compared to the model group. *Flammulina rossica* fermentation extract was rich in polysaccharide components and can bind to polysaccharide receptors on erythrocyte membranes. The effect of the dose on polysaccharide absorption was very complex and nonlinear. The complex pattern of polysaccharide nonlinear absorption may result from the reduced interaction of intestinal absorption and secretory transport systems when polysaccharide concentrations exceed those of linear absorption [30]. In this experiment, at a low dose, the binding effect of the red blood cells was the best, so the complement type I receptor (CR1) on the surface of the red blood cells was fully expressed on the membrane, and, finally, the ability of the red blood cells to immune-adhere to cells was enhanced and circulating immune complexes in the blood were removed [31].

### 2.7. Effect of FREP on Serum Antibody-Forming Cells

**Figure 6:**
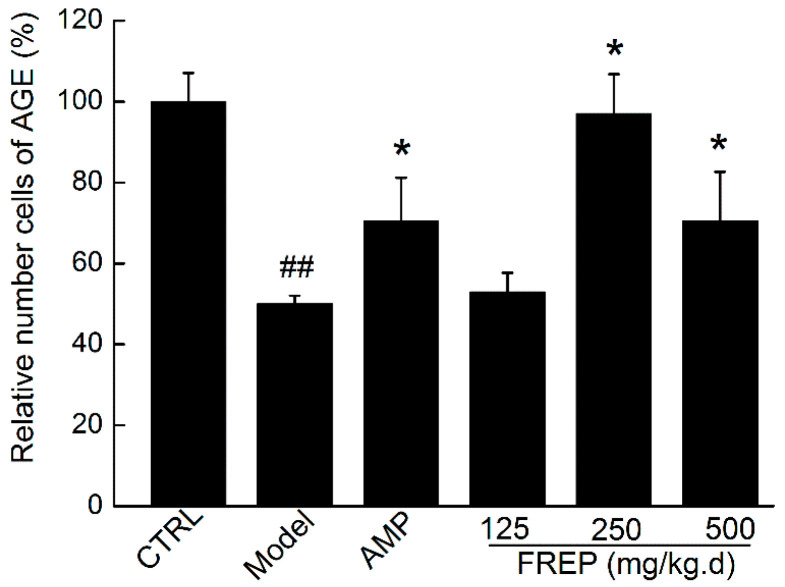
Effect of FREP on the number of antibody-forming cells in Cy-treated mice. The data were analyzed using one-way ANOVA and are expressed as means ± SEMs (*n* = 8). ## *p* < 0.01 in comparison with the control group; * *p* < 0.05 as compared with the model group. CTRL: normal control; Model: model group; AMP: *Astragalus membranaceus* polysaccharides; and FREP: *Flammulina rossica* fermentation extract.

Antibody-forming cells are an important indicator of humoral immunity status. To determine the effects of FREP on humoral immunity, the number of antibody-forming cells was measured by quantitative haemolysis spectrophotometry, and the results are shown in Figure 6. The relative number of antibody-forming cells in the model group was significantly (*p* < 0.01) lower than that in the normal group. However, the relative number of antibody-forming cells was significantly increased in the medium- and high-dose FREP groups (250 and 500 mg/kg/high) and the AMP group compared to the model group (*p* < 0.05, *p* < 0.05). The results suggested that FREP can enhance humoral immunity. 

### 2.8. Effect of FREP on Serum Haemolysin Formation

**Figure 7:**
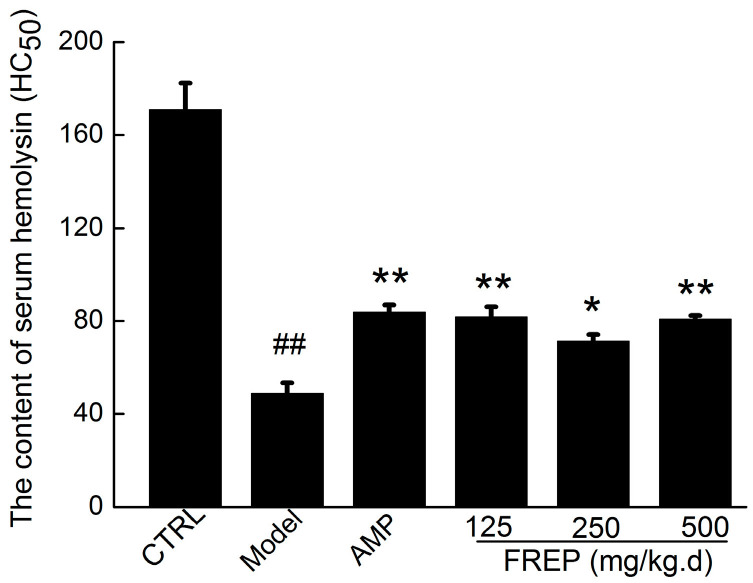
Effect of FREP on the content of serum hemolysin. The data were analyzed using one-way ANOVA and are expressed as means ± SEMs (*n* = 8). ## *p* < 0.01 in comparison with the control group; * *p* < 0.05 and ** *p* < 0.01 as compared with the model group. CTRL: normal control; Model: model group; AMP: *Astragalus membranaceus* polysaccharides; and FREP: *Flammulina rossica* fermentation extract.

To further investigate the effect of FREP on the humoral immune response, the serum haemolysin content was determined. As shown in Figure 7, the production of serum haemolysin was observably suppressed in the model control compared with the normal control (*p* < 0.05). Meanwhile, the serum haemolysin level was significantly increased in all three FREP-dose groups (125, 250, 500 mg/kg/high) and the AMP group compared to the model group. The results were consistent with previous reports [32,33]. Haemolysin is another indicator of humoral immunity status. Haemolysin is promoted after administration, suggesting enhanced humoral immunity after administration in the body [34,35]. Furthermore, the formation of serum haemolysin with SRBC immunization reflects the humoral immunologic function [27]. These findings further suggested that FREP can enhance humoral immunity.

### 2.9. Effect of FREP on Secretory Immunoglobulin A (SIgA) in Intestinal Contents

**Figure 8:**
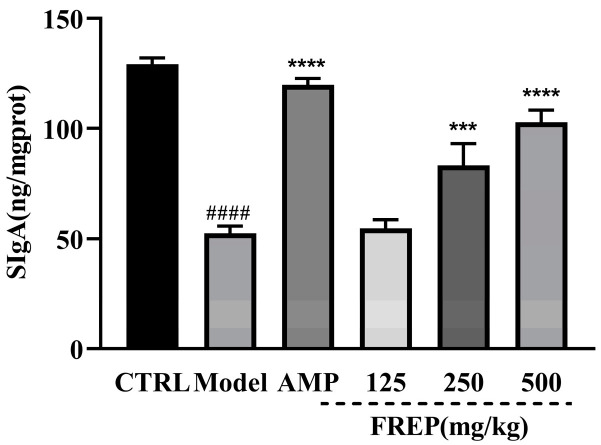
Effect of FREP on SIgA in intestinal contents. The data were analyzed using one-way ANOVA and are expressed as means ± SEMs (*n* = 8). #### *p* < 0.0001 in comparison with the control group; *** *p* < 0.001 and **** *p* < 0.0001 as compared with the model group. CTRL: normal control; Model: model group; AMP: *Astragalus membranaceus* polysaccharides; and FREP: *Flammulina rossica* fermentation extract.

Compared with the control group, SIgA in the model group was significantly decreased (*p* < 0.0001), and SIgA was significantly higher in the FREP group than in the model group (*p* < 0.0001) in a dose-dependent manner (Figure 8). The results showed that FREP could enhance the intestinal mucosal immunity of mice.

It was found that *Flammulina* species polysaccharides can promote the transformation function of spleen lymphocytes in healthy mice, enhance the activity of NK cells, increase the content of cytokine IL-2 in serum, enhance the phagocytosis of peritoneal macrophages, and increase the content of haemolysin in serum [36]. The study of the signal transduction pathway of immune enhancement is of great significance.

A large number of studies have shown that activation of the NF-κB signal transduction pathway is related to immune activity. NF-κB is a nuclear transcription factor of the Rel family that exists widely in vivo. To date, five members of the family have been found in mammalian cells: NF-κB p50, NF-κB p52, RelB, RelA (p65), and C-Rel can form homodimers or heterodimers and initiate the transcription of different genes [37]. At rest, the NF-κB dimer and inhibitory protein IκB combine into a trimer and hide in the cytoplasm. The ubiquitination degradation pathway of IκB can be activated by extracellular stimulation, which causes the NF-κB dimer to enter the nucleus, regulates the expression of immune-related cytokines and receptor genes, affects many biological functions of the body, and generally participates in many physiological and pathological pathways in the body [38].

## 3. Materials and Methods

### 3.1. Microorganism and Culture Conditions

*Flammulina rossica* was collected at Shanri-La in Yunnan, and the *Flammulina rossica* strain was screened and stored in our laboratory. The seed culture was grown in a 500 mL shake flask for 5 days at 150 rpm in a medium containing 200 g/L potato solution (200 g potato was boiled in water for 30 min, and the supernatant was kept for the medium), 20 g/L glucose, 1.5 g/L KH_2_PO_4_, 0.75 g/L MgSO_4_, and 0.001 g/L V_B1_. Liquid fermentation was performed in a 1000 mL shake flask containing 400 mL of medium inoculated with 10% (*v*/*v*) seed liquid. The liquid fermentation medium was composed of 20 g/L glucose, 40 g/L corn flour, 6 g/L yeast extract power, 1.5 g/L KH_2_PO_4_, 0.75 g/L MgSO_4_, and 0.1 g/L V_B1_. The shake flasks were incubated at 26 °C for 6 days. All media were sterilized at 121 °C for 30 min.

### 3.2. Preparation of the Fermentation Extract

The fermentation broths of *Flammulina rossica* were collected by gauze filter and concentrated to 1/10 of the original volume. The concentrated liquid was precipitated with four volumes of absolute ethanol for 12 h. Then, the sample was collected by centrifugation at 5000 rpm for 20 min and further freeze-dried and named FREP [39].

### 3.3. Measurement of the FREP Components

#### 3.3.1. Main Components

The total carbohydrate content in FREP was determined by the phenol-sulfuric acid colorimetric method as previously reported [40]. The reducing sugar was determined by the 3,5-dinitrosalicylic acid colorimetric method [41]. Protein content was measured with a bicinchoninic acid (BCA) assay [42]. The crude fat was determined by the petroleum benzine extraction method [43].

#### 3.3.2. Amino Acids

The FREP was hydrolyzed using 6 mol/L HCl at 110 °C for 22 h. After vacuum drying, the sample was dissolved in 1 mL buffer (pH 2.2). The quantitative analysis of the amino acids was carried out according to the method described previously [44].

### 3.4. FTIR Spectra Analysis

One milligram of dried FREP fermentation extract was mixed with dried potassium bromide (KBr) powder (190 mg) and then pressed into tablets at 4000–400 cm^−1^ (Nicolet 5700, Thermo Scientific, Waltham, MA, USA).

### 3.5. Molecular Weight

GPC-RI-MALS (Gel Chromatography-Differential Analysis-Multi-Angle Laser Light Scattering) was used to detect the molecular weight distribution of samples. Five milligrams of the FREP sample were dissolved in 1 mL of mobile phase at 45 °C. The mixture was centrifuged at 14,000 rpm for 10 min, and 100 μL of supernatant was measured by GPC-RI-MALS (DAWN HELEOSII, Wyatt Technology, Santa Barbara, CA, USA). The detection system included an Agilent 1260 HPLC system (Agilent, Palo Alto, CA, USA), an Optilab T-rEX refractive index detector (Wyatt Technology, CA, USA), and three analytical columns composed of Ohpak SB-805 HQ (300 mm × 8 mm), Ohpak SB-804 HQ (300 mm × 8 mm), and Ohpak SB-803 HQ (300 mm × 8 mm) (Shodex, Asahipak, Tokyo, Japan). The mobile phase was 0.1 mol/L of NaNO_3_ solution at a flow rate of 0.4 mL/min [45].

### 3.6. Monosaccharide Composition of the FREP

The monosaccharide composition was determined by high-performance liquid chromatography (HPLC) [46]. The polysaccharide samples were dialyzed using 3500 kDa dialysis bags to remove small molecules and then freeze-dried. An amount of 2 mg of the freeze-dried sample was weighed into a sealed vial, 1 mL of the substance was added to a 1 mol/L methanol hydrochloric acid solution, and the flask was sealed with nitrogen. After hydrolysis in a constant temperature drying oven at 80 °C for 16 h, the liquid in the flask was blown dry with an air pump. Then, 1 mL of 0.5 mol/L trifluoroacetic acid solution was added to the vial and heated in a 120 °C constant temperature drying oven for 1 h. The liquid in the flask was transferred to an evaporation dish, and ethanol was continuously added to it to evaporate the residual trifluoroacetic acid. Finally, the reaction solution was dried. First, 0.5 mL of PMP (1-phenyl-3-methyl-5-pyrazolone) was added to the acid hydrolysates of the fermentation extract samples. The methanol solution was added to 0.5 mL 0.3 mol/L sodium hydroxide solution, shaken well, and then heated in a water bath at 70 °C for 30 min. The samples were centrifuged at 4000 rpm × 3 min, and 50 μL of 0.3 mol/L hydrochloric acid was added to the upper natant. The solution and 50 μL distilled water were mixed, and then 1 mL chloroform was added. After shaking, the solution was centrifuged at 10,000 rpm × 3 min to collect the upper aqueous solution. The samples were filtered through a 0.22 μm filter and detected by HPLC. The instrument was a Shimadzu HPLC system, the elution flow rate was 1.0 mL/min, the chromatographic column was a Thermo ODS HYPERSIL column (4.6 mm × 150 mm), and the eluate was 81.8% PBS solution (0.1 mol/L, pH 7.0) and 18.2% acetonitrile (*v*/*v*). The detection temperature was 35 °C, and the detection wavelength was 245 nm.

### 3.7. Animals

Kunming male mice (4–5 weeks old, 19–23 g) were purchased from the Medical Laboratory Animal Center of Chang Chun Gao Xin. The mice were fed water and mouse chow ad libitum and were housed in a rodent facility at 22 ± 1 °C with a 12 h light–dark cycle for acclimatization.

### 3.8. The Effects of FREP on Healthy Mice

**Figure 9:**
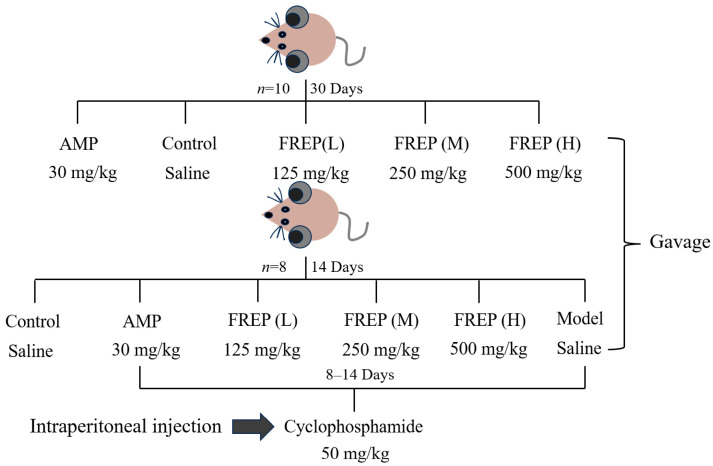
Mouse modeling process.

The mice were randomly divided into 5 groups composed of 10 mice each [47]. The animals were administered as follows: the positive control group was administered 30 mg/kg body weight (BW) *Astragalus membranaceus* polysaccharides (AMP), the normal control group was administered physiological saline, and the three drug groups were administered 125, 250, and 500 mg/kg body weight (BW) FREP. All these treatments were administered intragastrically one time daily for 30 days. The modeling process is shown in Figure 9.

### 3.9. The Effects of FREP on Immunosuppressive Mice

The mice were randomly separated into 6 groups composed of 8 mice each. One group of healthy mice was used as the normal control group and administered physiological saline; the positive control group was administered 30 mg/kg BW *Astragalus membranaceus* polysaccharides (AMP), the model control group was administered physiological saline, and the three drug groups were administered 125, 250, and 500 mg/kg body weight (BW) FREP. All these treatments were administered intragastrically one time daily for 14 days. On the eighth day, the AMP group, the model control group, and the low-, medium-, and high-dose FREP-treatment groups were injected with cyclophosphamide (Cy) at 50 mg/kg BW/d by means of intraperitoneal injection on three consecutive days. The moulding process is shown in Figure 1.

### 3.10. Immune Organ Index Measurement

After the mice were anaesthetized with ether and euthanized, they were soaked in 75% ethanol for 3–5 min and dissected on a sterile operating table. The thymus and spleen of healthy mice and immunosuppressive mice were collected, and the surrounding fat was removed and weighed to calculate the thymus and spleen index.

### 3.11. Spleen Cell Proliferation Experiment

#### 3.11.1. Preparation of Spleen Cells

The extirpated spleens were treated under aseptic conditions. Then, the spleens were filtered through a 200-sieve mesh and ground with a needle core. Samples were washed with serum medium and centrifuged at 3000 r/min for 5 min at 4 °C. The spleen cells were resuspended and adjusted to a concentration of 2 × 10^6^ cells/mL with RPMI-1640 medium supplemented with 10% foetal calf serum.

#### 3.11.2. Effect of FREP on Spleen Lymphocyte Proliferation

Then, 100 μL splenocytes were added to 96-well plates, and the experimental groups were supplemented with 100 μL RPMI-1640 medium containing 5 μg/mL concanavalin A (Con A) and different doses of FREP (5–1000 μg/mL). Equal volumes of RPMI-1640 medium and RPMI-1640 medium containing only Con A (5 μg/mL) were added to the control group and Con A group, respectively. The microplates were incubated at 37 °C in a 5% CO_2_ incubator for 48 h. Then, 20 μL of 5 mg/mL MTT solution was added to each well and cultured for 4 h. Then, the cell suspensions were discarded, and 200 μL of DMSO was added. The absorbance value of splenocyte cells was determined with a microplate reader (Bio-Rad, Hercules, CA, USA) at 570 nm [48,49]. The absorbance value of the normal group was set as 100%, and the relative proliferation of the other groups was calculated.

### 3.12. Effects of FREP on CD^4+^ and CD^8+^ T Lymphocytesin Healthy Mice

The splenocyte suspension was incubated for 1 h at 4 °C in dark conditions with 10 μL of CD4-PE or CD8-APC. Then, the cells were washed with PBS and resuspended in 1% paraformaldehyde. The counts of CD^4+^ and CD^8+^ T lymphocytes were measured by flow cytometry (Becton Dickinson, Accuri C6, Franklin Lakes, NJ, USA) [29].

### 3.13. Effects of FREP on Peripheral White Blood Cell and Red Blood Cell Counts in Immunosuppressive Mice

At the end of the experiment on the effect of FREP on immunosuppressive mice, the blood of all animals was collected by retro-orbital bleeding into heparin tubes. The platelet counts of white blood cells (WBCs) and red blood cells (RBCs) were analyzed using a cell counter.

### 3.14. Determination of Cytokines in Serum

Serum was collected by enucleating the orbital sinus after the last administration of FREP to healthy mice, and the concentrations of IL-2, TNF-α, IL-8, IL-10, IL-12, IFN-γ, IL-1β, and IL-6 were measured using ELISA kits (Jingmei, Jiangsu, China). The ELISA kit we selected had intra-assay CV values within 10% and inter-assay CV values within 15%. Following are the sensitivity of ELISA kits for different indicators (Table 4). 

### 3.15. Measurement of Serum Haemolysin

The serum haemolysin level was determined according to a previous report [50,51]. The grouping and feeding of the mice were the same as described in Section 3.8 and Section 3.9. After the ninth day of FREP administration, each animal was immunized by injection of sheep erythrocytes (SRBC, 10%). After five days, the mice were sacrificed, and blood samples were collected. The serum was isolated and diluted 200 times with PBS. Then, 1 mL of complement (1:10 dilution) and 0.5 mL of 5% SRBC were mixed with 1 mL of diluted serum. The mixed sample was incubated for 1 h at 37 °C and was immediately moved to an ice bath and centrifuged at 2000 rpm for 10 min. Approximately 1 mL of supernatant was mixed with 3 mL of Drabkin’s solution for 10 min. Then, the absorbance value was measured at 540 nm and recorded as A. Another 0.25 mL of 5% SRBC was added to 4 mL of Drabkin’s solution, and the absorbance value was recorded as B and measured at the same wavelength. The half haemolytic value (HC 50) was calculated as follows: HC 50 of the sample = A/B.

### 3.16. Antibody-Forming Cells

The number of antibody-forming cells was determined with quantitative haemolysis spectrophotometry (QHS) [32]. The grouping and feeding of the mice were the same as described in Section 3.8 and Section 3.9. After the sixth day of FREP administration, each mouse was primarily immunized by injection of a 0.2 mL suspension of sheep erythrocytes (SRBC, 5%), except for the normal control group. After three days, secondary immunization was performed with the same method. Four days after immunization, the mice were sacrificed and spleen cells were prepared. Then, 0.5 mL of complement (1:10 dilution) and 0.2 mL of 5% SRBC were mixed with 1 mL of diluted serum. The mixed sample was incubated for 1 h at 37 °C, immediately moved to an ice bath, and centrifuged at 3000 rpm for 5 min. The supernatant was determined by a microplate reader at 413 nm.

### 3.17. Detection of Secretory Immunoglobulin A in Intestinal Contents

After weighing, the tissue was shredded, and 9 times the volume of normal saline was added to the shredded tissue to prepare a 10% intestinal homogenate. Then, the samples were centrifuged at 3500 r/min for 10 min, and the supernatant was collected for detection. The expression level of SIgA in the intestinal homogenate was detected by ELISA (Shanghai Langdun, Shanghai, China). The ELISA kit we selected had intra-assay CV values within 10% and inter-assay CV values within 15%. The sensitivity of ELISA kits was 0.3 pg/mL. 

### 3.18. Statistical Analysis

All of the numerical experiment data are expressed as mean ± standard deviation (SD). The statistical analyses were carried out with the SPSS 17.0 (IBM Company, Chicago, IL, USA), Origin (Origin Lab Corporation, San Mateo, CA, USA) and GraphPad Prism 8 (Graphpad Company, San Diego, CA, USA) software package.

## 4. Conclusions

The FREPs were prepared from the fermentation broths of *Flammulina rossica*. The present study has demonstrated that FREP consists of mannose, glucose, galactose, arabinose, and fucose and has a β-glycosidic bond. The molecular weight of FREP was 28.52 kDa. It not only promoted the activation of T and B cells in healthy mice but also improved the immune functions in immunosuppressive mice. The results suggest that FREP could be developed as an immunomodulatory agent and potential immunotherapeutic medicine for patients with inadequate immune function.

## Figures and Tables

**Table 1:** Composition and physicochemical characteristics of FREP.

Sample	FREP (%)
Total carbohydrate	40.5
Total protein	10.4
Reducing sugar	16.67
Crude fat	0.48
Molecular weight (kDa)	28.52
Monosaccharied composition (mol %)	
Mannose	22.4
Glucose	26.5
Galactose	37.7
Arabinose	12.3
Fucose	1.1

**Table 2:** Percentage composition of amino acids in FREP.

Compounds	Contents (%)	Compounds	Contents (%)
Aspartic acid (Asp)	0.18	Isoleucine (Iso)	0.13
L-Threonine (Thr)	0.16	Leucine (Leu)	0.20
Serine (Ser)	0.22	Tyrosine (Tyr)	0.10
Glutamic acid (Glu)	1.12	Phenylalanine (Phe)	0.12
Glycine (Gly)	0.30	Lysine (Lys)	0.15
Alanine (Ala)	0.18	Histidine (His)	0.06
Valine (Val)	0.15	Arginine (Arg)	0.19
DL-Methionine (Met)	0.07	Proline (Pro)	0.16

**Table 3:** Effect of FREP on the lymphocyte subpopulation in healthy mice.

Group	Dosage (mg/kg/d)	CD^4+^ (%)	CD^8+^ (%)	CD^4+^/CD^8+^
Control		28.20 ± 0.93	17.60 ± 0.47	1.62 ± 0.024
AMP	30	80.52 ± 6.25 **	11.83 ± 0.29 **	2.67 ± 0.075 **
FREP	500	31.50 ± 0.38 *	14.17 ± 0.22 **	2.23 ± 0.055 **
FREP	250	33.50 ± 0.38 **	17.67 ± 0.29	1.89 ± 0.012 **
FREP	125	31.60 ± 0.68 *	19.50 ± 0.45 *	1.62 ± 0.010

* *p* < 0.05 and ** *p* < 0.01 as compared with the normal control group.

**Table 4:** The sensitivity of ELISA kits for different indicators.

Indicators	Sensitivity (pg/mL)
IL-2	7.5
TNF-α	6.25
IL-8	2
IL-10	7.5
IL-12	0.125
IFN-γ	7.5
IL-1β	0.625
IL-6	0.75

## Data Availability

Not applicable.

## Supplementary Materials

The following are available online at https://www.mdpi.com/article/10.3390/molecules28155825/s1, Figure S1: Results of ITS sequence alignment.

## Sample Availability

Samples of the compounds are not available from the authors.
