# Supplementary material for: Immunomodulatory Effect of Flammulina rossica Fermentation Extract on Healthy and Immunosuppressed Mice
Source: Molecules. 2023 Aug 2;28(15):5825. doi: 10.3390/molecules28155825 (PMC10421243; doi:10.3390/molecules28155825)
Supplement: Supplementary file 1 [file molecules-28-05825-s001.zip › molecules-2470194-supplementary.pdf]

Sequence ID: KC179735.1 Length: 796 Number of Matches: 1

Range 1: 39 to 796 GenBank Graphics ▼ Next Match ▲ Previous Match

Score	Expect	Identities	Gaps	Strand
1310 bits(709)	0.0	746/762(98%)	10/762(1%)	Plus/Plus

```
Query  3      TTG-ACGCTTGTGGCTTCAGGCTGTTGCTGACGGGACCTTCACGGGTTTCGTACGTGCAC  61
              ||| ||||||||||||||||||||||||||||||||||||||||||||||||||||||||
Sbjct  39     TTGAACGCTTGTGGCTTCAGGCTGTTGCTGACGGGACCTTCACGGGTTTCGTACGTGCAC  98

Query  62     GTCTGAGTTGCAACTTTCTTCGTCCACCTGTGCACACTCTGTAGGTCTGGATAACCCCA-  120
              ||||||||||||||||||||||||||||||||||||||||||||||||||||| ||||| 
Sbjct  99     GTCTGAGTTGCAACTTTCTTCGTCCACCTGTGCACACTCTGTAGGTCTGGATA-CCCCAT  157

Query  121    TTTGAAGGGTGCACGTC-TTCAAAAGCGCGCTCCCTTTGCCTTCCAGGCCTATGTCTTAT  179
              ||||||||||||||||| || |||||||||||||||||||||||||||||||||||||||
Sbjct  158    TTTGAAGGGTGCACGTCTTTAAAAAGCGCGCTCCCTTTGCCTTCCAGGCCTATGTCTTAT  217

Query  180    AAACACTTTTGTATGTAATGAATGTCATTGATTATTGGACCTCACCGTCCTTTAAACTAA  239
              |||||||||||||||||| |||||||||||||||||||||||||||||||||||||||||
Sbjct  218    AAACACTTTTGTATGTAACGAATGTCATTGATTATTGGACCTCACCGTCCTTTAAACTAA  277

Query  240    ATACAACTTTCAACAACGGATCTCTTGGCTCTCGCATCGATGAAGAACGCAGCGAAATGC  299
              ||||||||||||||||||||||||||||||||||||||||||||||||||||||||||||
Sbjct  278    ATACAACTTTCAACAACGGATCTCTTGGCTCTCGCATCGATGAAGAACGCAGCGAAATGC  337

Query  300    GATAACTAATGTGAATTGCAGAATTCAGTGAATCATCGAGTCTTTGAACGCACCTTGCGC  359
              ||||||||||||||||||||||||||||||||||||||||||||||||||||||||||||
Sbjct  338    GATAACTAATGTGAATTGCAGAATTCAGTGAATCATCGAGTCTTTGAACGCACCTTGCGC  397

Query  360    CCTTCGGCACTCCGAAGGGCATGCCTGTTTGAGTGTCAGTAACTTCTCAACCTCCCTCAC  419
              |||| |||||||||||||||||||||||||||||||||||||||||||||||||||||||
Sbjct  398    CCTTTGGCACTCCGAAGGGCATGCCTGTTTGAGTGTCAGTAACTTCTCAACCTCCCTCAC  457

Query  420    TTTGTTGTGAGCTGGCGGATTGGATGGTGGGGGCTTGCTGGACCTCTCTTGGTTCAGCTC  479
              |||| |||||||||||||||||||| ||||||||||||||||||| ||||||||||||||
Sbjct  458    TTTGCTGTGAGCTGGCGGATTGGAT-GTGGGGGCTTGCTGGACCTATCTTGGTTCAGCTC  516

Query  480    CCCTGAAATGCATTAGCAGAAACCGTTACCTTTTGGCGCGCTGCAGCTGTGATAATTATC  539
              ||||||||||||||||||||||||||||||||||||||||||||||||||||||||||||
Sbjct  517    CCCTGAAATGCATTAGCAGAAACCGTTACCTTTTGGCGCGCTGCAGCTGTGATAATTATC  576

Query  540    TACGGCTATGGCTGGGCTGACTGTGTTGTAGCGCTTGTTCGTCTCTGAAGGGTTTTGCCC  599
              |||||||||||||||||||||||||||||||||||||||||||||||||||| |||||||
Sbjct  577    TACGGCTATGGCTGGGCTGACTGTGTTGTAGCGCTTGTTCGTCTCTGAAGGG-TTTGCCC  635

Query  600    TAGTGGCCTTCCCTTTGCCTTCTCTCTCACGAGAGATACCTGTGACGCGAGTGCGCGGGC  659
              ||||||||||||||||||||||||| ||||||||||||||||||||||||||||||||||
Sbjct  636    TAGTGGCCTTCCCTTTGCCTTCTCTTTCACGAGAGATACCTGTGACGCGAGTGCGCGGGC  695

Query  660    TATTCAGCTTCTAACCGTCCCTTTGGGGACAACTTATTGACCATTTGACCTCAAATCAGG  719
              ||||||||||||||||||||||||||||||||| ||||||||||||||||||||||||||
Sbjct  696    TATTCAGCTTCTAACCGTCCCTTTGGGGACAAC-TATTGACCATTTGACCTCAAATCAGG  754

Query  720    TAGGACTACCCGCTGAACTTAAGCATATCA-TAA-CGG-GGA  758
              |||||||||||||||||||||||||||||| ||| ||| |||
Sbjct  755    TAGGACTACCCGCTGAACTTAAGCATATCAATAAGCGGAGGA  796
```

Figure S1. Results of ITS sequence alignment (Compared with *Flammulina rossica* GenBank: KC179735.1 on NCBI, the similarity was over 99%, and it was identified as *Flammulina rossica*.)
